# MMP-Inhibitory Effects of Flavonoid Glycosides from Edible Medicinal Halophyte* Limonium tetragonum*

**DOI:** 10.1155/2017/6750274

**Published:** 2017-09-20

**Authors:** Min Joo Bae, Fatih Karadeniz, Jung Hwan Oh, Ga Hyun Yu, Mi-Soon Jang, Ki-Ho Nam, Youngwan Seo, Chang-Suk Kong

**Affiliations:** ^1^Department of Food and Nutrition, College of Medical and Life Sciences, Silla University, 140, Baegyang-daero 700beon-gil, Sasang-gu, Busan 46958, Republic of Korea; ^2^Marine Biotechnology Center for Pharmaceuticals and Foods, Silla University, 140, Baegyang-daero 700beon-gil, Sasang-gu, Busan 46958, Republic of Korea; ^3^Food Safety and Processing Research Division, National Institute of Fisheries Science, Busan 46083, Republic of Korea; ^4^Division of Marine Bioscience, Korea Maritime and Ocean University, Busan 49112, Republic of Korea; ^5^Ocean Science and Technology School, Korea Maritime and Ocean University, Busan 49112, Republic of Korea

## Abstract

*Limonium tetragonum* has been well-known for its antioxidative properties as a halophyte. This study investigated the antimetastasis effect of solvent-partitioned* L. tetragonum* extracts (LTEs) and isolated compounds on HT1080 mouse melanoma cell model with a focus on matrix metalloproteinase (MMP) activity and TIMP and MAPK pathways. Upregulation and stimulation of MMPs result in elevated degradation of extracellular matrix which is part of several complications such as metastasis, cirrhosis, and arthritis. The anti-MMP capacity of LTEs was confirmed by their MMP-inhibitory effects, regulation of MMP and TIMP expression, and suppression of MAPK pathway. Among all tested LTEs, 85% aq. MeOH and n-BuOH were found to be most active fractions which later yielded two known flavonoid glycosides, myricetin 3-galactoside and quercetin 3-o-beta-galactopyranoside. Anti-MMP potential of the compounds was confirmed by their ability to regulate MMP expression through inhibited MAPK pathway activation. These results suggested that* L. tetragonum* might serve as a potential source of bioactive substances with effective anti-MMP properties.

## 1. Introduction

Spread and growth of malignant tumorous cells are conducted through invasive and metastatic activities of cancer cells. Evolution of a primary abnormal tissue growth from a neoplasm to invasive tumor cells includes several-step biological processes. Degradation of extracellular matrix with proteolytic enzymes is one of the main pathways regulating the aforementioned evolution [1]. Matrix metalloproteinases (MMPs), zinc-containing calcium-dependent endopeptidases, are responsible for the proteolytic degradation of extracellular matrix and hence closely linked with tumor invasion, angiogenesis, and metastasis of cancer. In invasive tumor cells, two MMPs, MMP-2 and MMP-9, are predominantly upregulated and take crucial roles in invasion and metastatic spread of tumor [2, 3]. Regulation of MMP activities is carried out by intracellular inhibitors called tissue inhibitor of metalloproteinases (TIMPs) of which TIMP-1 and TIMP-2 are studied intensely in cancer studies. In this context, balance between MMPs and TIMPs was suggested to be deteriorated during cancer spread and growth which promotes a new target for therapeutic action against malignant tumors. Inhibition of MMPs coupled with increase in TIMP activity is also considered a promising path against tumor cell growth [4]. In addition, therapeutic intervention in metastasis and growth of tumor cells is aimed to be achieved by natural agents which lately are sourced from medicinal plants and traditional folk medicine [5–7]. The scientific world witnessed discovery and development of novel and potential bioactive substances against cancer from natural sources, especially both terrestrial and marine plants [8–10]. Recent developments in natural product research suggests that plants are important sources that exhibit anticancer properties through MMP/TIMP regulation tumor cells.

Salt marshes and muddy seashores of South Korea contain a commonly known edible halophyte species,* Limonium tetragonum*, which grows in places that come into contact with waters of high salinity [11]. However, apart from antioxidants which are known to be present in abundance in plants of harsh environments, current studies do not contain any detailed report on health beneficial effect of* L. tetragonum* [12]. Considering the potential of halophytes, as a part of prominent research trend to develop novel substances from plants for nutraceutical purposes, solvent-partitioned* L. tetragonum* extracts has been tested.* L. tetragonum* was assayed for its effect on MMP-linked pathways* in vitro* using a human fibrosarcoma cell model. Isolated bioactive substances that have potential use against MMPs were characterized and screened.

## 2. Materials and Methods

### 2.1. Plant Material

*L. tetragonum *was provided by Korea Maritime and Ocean University (Yeongdo, Busan, Korea). Crude extracts were prepared as described by Bae et al. [13]. Solvent fractions were obtained from the organic and aqueous layers. First, CH_2_Cl_2_ layer was fractionated with n-hexane and 85% aqueous MeOH. The aqueous layer was further fractionated with n-BuOH and H_2_O, resulting in* L. tetragonum* solvent-partitioned fractions (LTEs) of n-hexane (2.64 g), 85% aqueous (aq.) MeOH (1.42 g), n-BuOH (1.53 g), and H_2_O (26.47 g). Fractions were dissolved in 10% DMSO in order to be used in experiments. Further isolation of the active compounds was carried out as described earlier [14].

### 2.2. Cell Culture and Cytotoxicity Determination

HT1080 human fibrosarcoma cell line was used for* in vitro* assays. Cells were grown in flasks (T-75, Nunc, Roskilde, Denmark) at 37°C humidified atmosphere of 5% CO_2_ and fed with Dulbecco's modified Eagle medium (DMEM, Gibco-BRL, Gaithersburg, MD, USA) containing 10% fetal bovine serum (FBS), 2 mM glutamine, and 100 *μ*g/mL penicillin-streptomycin (Gibco-BRL, Gaithersburg, MD, USA). Culture medium was replaced with fresh one every 3 days unless otherwise stated.

For cell viability assessment, cells were cultured in 96-well plates at 5 × 10^3^ cells/well density. Following 24 h incubation, cell culture medium was removed and the cells were washed with fresh medium prior to treatment with samples (5, 10, 20, 50, and 100 *μ*g/mL). Cells were rewashed with fresh medium after 24 and 48 h of incubation and 100 *μ*L of MTT solution (1 mg/mL) was introduced to the wells, followed by a 4 h incubation. Finally, 100 *μ*L of DMSO was used for each well in order to solubilize the formazan crystals for the determination of absorbance values at 540 nm using a GENios® microplate reader (Tecan Austria GmbH, Grödig, Austria). Cell viability was defined by the absorbance value as a way to indicate the amount of MTT converted into formazan crystal. Viability of cells was determined by comparing untreated control against sample treated wells and dose-response curves were established as percentage.

### 2.3. Determination of MMP Activity by Gelatin Zymography

Enzymatic activities of MMP-2 and MMP-9 from HT1080 cells that were treated with or without samples were detected by gelatin zymography. Method of Bae et al. [13] was followed with small modifications. Briefly, phorbol 12-myristate 13-acetate (PMA, 10 ng/mL) was used to enhance the MMP expression of cells and conditioned cell culture media were used to determine the activity of MMPs following treatment with LTEs or isolated compounds at the stated concentrations. Following substrate-gel electrophoresis of cell culture medium, gels were kept in a buffer solution containing 10 mM CaCl_2_, 50 mM Tris–HCl, and 150 mM NaCl at 37°C for 48 h in order to promote the gelatin digestion of MMPs. Activities of MMP were observed as digested clear zones following subjection to Coomassie Blue staining under a CAS-400SM Davinch-Chemi imager™ (Davinch-K, Seoul, Korea).

### 2.4. RNA Isolation and Reverse Transcription-Polymerase Chain Reaction (RT-PCR) Analysis

Effects of samples on mRNA expression of MMPs and TIMPs were analyzed by RT-PCR. Common protocols were followed using the sense and antisense primers that were stated previously [13]. Briefly, 2 *μ*g of total RNA from treated or untreated cells was converted to single stranded cDNA using a reverse transcription system (Promega, Madison, WI, USA) and later amplified using specific primers according to manufacturer's protocol. Final products were visualized under UV light using a CAS-400SM Davinch-Chemi imager™ (Davinch-K, Seoul, Korea) and AlphaEase® gel image analysis software (Alpha Innotech, San Leandro, CA, USA) was used for quantification.

### 2.5. Western Blot Analysis

Immunoblotting was performed according to common standard procedures described earlier [13]. To explain briefly, HT1080 cells were agitated in RIPA lysis buffer (Sigma-Aldrich Corp., St. Louis, USA) at 4°C for 30 min. Cell lysates (35 *μ*g) were subjected to electrophoresis using 12% SDS-polyacrylamide gel for separation which was followed by transfer onto a polyvinylidene fluoride membrane (Amersham Pharmacia Biosciences, England, UK). Detection of immunoreactive proteins was carried out with an electrochemiluminescence kit (Amersham Pharmacia Biosciences, England, UK). Protein bands on membranes were observed using a CAS-400SM Davinch-Chemi imager™ (Davinch-K, Seoul, Korea).

### 2.6. Statistical Analysis

All plotted data were presented as a mean of three different experiments ± SD. Differences between the calculated means of each individual group were determined by one-way ANOVA coupled with Duncan's multiple range tests using the statistical software SAS v9.1 (SAS Institute, Inc., Cary, NC, USA). Any difference was considered statistically significant at *P* < 0.05.

## 3. Results and Discussion

MMPs are known to influence and intervene with several particular important pathways for metastasis, oxidative stress, and fibrosis [15]. Hence, successful inhibition of MMP activity steadily gains high interest to develop potent pharmaceuticals against metastasis-linked complications and tumor growth. MMP inhibitors of natural origin are being intensively studied nowadays and marine organisms hold a great deal of potential in this context due to their ability to survive in unique and challenging environments. Various organisms, especially marine plants, are promoted to contain bioactive metabolites some of which were credited as potent MMP inhibitors with suggested mechanism of actions [16–18]. In order to provide valuable insights on that matter,* L. tetragonum* was studied to evaluate its MMP-inhibition efficiency along with MMP inhibiting constituents. In this regard, extract of* L. tetragonum *was fractioned with organic solvents and solvent-partitioned extracts were tested separately. As a part of ongoing research, previously reported MMP-inhibitory activities of crude extract of* L. tetragonum *[13] were further detailed in order to provide the action mechanism and rationale behind the findings by characterization of active constituents.

**Figure 1:**
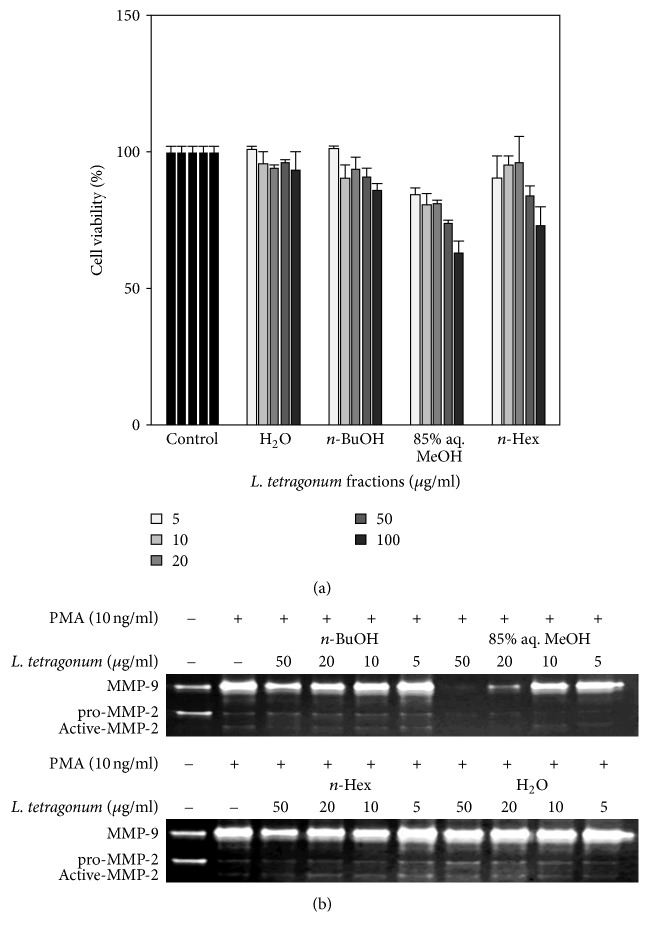
(a) Effect of solvent-partitioned* L. tetragonum* extracts (LTEs) on cell viability of HT1080 human fibrosarcoma cells. (b) Effect of LTEs on enzymatic activity of MMP-2 and MMP-9 tested by gelatin zymography. Values are mean ± SD (*n* = 3).

First, the LTE samples were tested for their cytotoxic presence in human fibrosarcoma cell line HT1080 for 48 h at five different concentrations (5, 10, 20, 50, and 100 *μ*g/mL) (Figure 1(a)). The cytotoxicity test revealed that these concentrations were cytocompatible up to 50 *μ*g/mL and any observed inhibition of MMP-2 and MMP-9 activity was not caused by any cytotoxic influence at the concentrations below that.

LTEs were analyzed for their possible activity to inhibit MMP-2 and MMP-9 enzymes following a PMA stimulation. Gelatinolytic activity of MMP-2 and MMP-9 secreted from fibrosarcoma cell line HT1080 was evaluated with gelatin zymography which was carried out using conditioned medium of LTE treated cells after PMA stimulation (Figure 1(b)). Introduction of PMA (10 ng/mL) to cells resulted in enhanced activation of MMP-2 and MMP-9; hence gelatinolytic activity in gelatin zymography was elevated. Among tested samples, 85% aq. MeOH LTE decreased the most of MMP-2 and MMP-9 activity in a dose-dependent manner relevantly higher than that of other samples. Remaining LTEs were observed to inhibit both MMP activity in an order of n-Hex, n-BuOH, and H_2_O fractions in regard to their efficiency. Inhibition of MMP enzymatic activities indicated that active samples possess bioactive substances that could have inhibit the MMP-2 and MMP-9 activity in the extracellular matrix dependent or independent from their intracellular secretion pathways. However, as seen in cell viability assay results (Figure 1(a)), the 85% aq. MeOH and n-Hex fractions exhibit increased cytotoxicity as the concentration increases. Although the enzymatic activity inhibition of LTEs was carried out using 50 ug/mL concentration, any possible inhibition may be caused by small amounts of cytotoxic substances in samples. However, at lower concentrations such as 10 and 20 ug/mL which at LTEs did not exhibit any cytotoxicity, an inhibition of both MMP-2 and MMP-9 activity still could be observed. In this context, further cell-based experiments were carried out with normalization of the results against a housekeeping gene in order to eliminate the any possible interference of cytotoxic presence.

**Figure 2:**
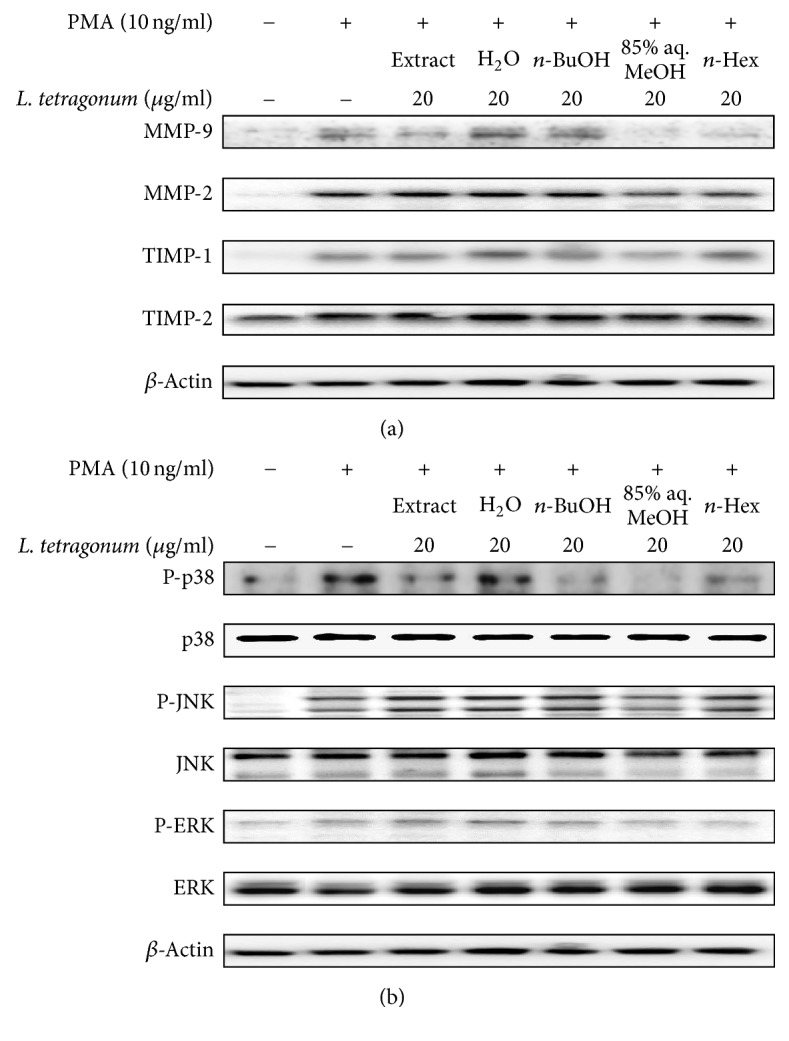
Effect of solvent-partitioned* L. tetragonum* extracts (LTEs) on protein levels of MMP-2 and MMP-9, TIMP-1 and TIMP-2, p38, JNK, and ERK observed by immunoblotting. *β*-Actin was used as an internal standard.

Further, protein levels of MMP-2 and MMP-9 were determined by immunoblotting along with the levels of TIMP-1 and TIMP-2. TIMPs are known inhibitors of MMPs and also reported to elevate the activity of MMP-2 in some situations as a part of regulation [19]. Immunoblotting results suggested the treatment with 20 *μ*g/ml LTEs was able to suppress the protein levels of MMP-2 and MMP-9 (Figure 2(a)). Presence of TIMPs is considered to imply inhibited MMP activity as a part of cellular response to extracellular stimuli [4]. Hence, the PMA stimulation caused TIMP levels to decrease and MMP expression to increase. However, treatment with LTEs was observed to produce mixed results regarding the effect on the TIMP levels following the PMA stimulation. Expected results were to inhibit MMP and enhance TIMP expressions in order to regulate the extracellular matrix degradation. According to results, only n-BuOH and n-Hex LTE were able to regulate the MMP-2, MMP-9, TIMP-1, and TIMP-2 levels in an expected manner where suppression of MMP expression is coupled with elevated TIMP levels. On the other hand, protein levels of MMP-2 and MMP-9 were slightly elevated after H_2_O and n-BuOH LTE treatment with elevated TIMP-1 and TIMP-2 levels. Elevated levels of TIMP-2 were observed, in addition to an increase in MMP-2 protein levels after n-Hex LTE treatment. Nonetheless, LTEs were shown to have effect on both activity and expression of MMP pathways but with suggested different mechanism of actions. In cases of H_2_O and n-BuOH LTEs, a possible intervention for the activation of MMP-2 and MMP-9 enzymes was suggested following the elevated protein levels of MMPs which would explain the inhibited enzyme activity despite the elevated protein levels. In terms of remaining LTEs, a mechanism where there isTIMP-linked regulation of MMP activity was suggested.

In order to grasp the suggested intervention on MMP-related intracellular pathways, levels of MAPK-related proteins, namely, p38, p-ERK, and p-JNK, were also evaluated (Figure 2(b)). Expectedly, PMA stimulation resulted in an elevated expression of ERK, JNK, and p-38 as well as their phosphorylation in regard to raised cellular activity. Reports showed that the inhibition of MAPK pathways is closely related to downregulation of MMP secretion [20, 21]. The activation of MAPK pathway induced by PMA stimulation was seen in Figure 2. However, treatment with LTEs at the concentration of 20 *μ*g/mL did not show any consistent change in aforementioned increment in protein expressions. Among tested samples, 85% aq. MeOH and n-BuOH were able to regulate the MAPK pathways besides the MMP and TIMP levels. Also, n-Hex and H_2_O LTE were not able show any notable effect on MAPK pathway. These results implied the suggested action mechanisms of LTEs. On the other hand, 85% aq. MeOH and n-BuOH LTEs were able to present a regulatory effect on MMP activation through suppression of MAPK pathway shown as lowered p-ERK, p-JNK, and p-38 levels. However, among all tested MAPK proteins, phosphorylated p38 levels were inhibited by treatment of all LTEs. Kim et al. [22] reported that upregulation of MMP-2 and MMP-9 was induced by TGF-*β*-linked p38 expression but not ERK and JNK signaling in human breast epithelial cells. Likewise, current data suggested that LTEs treatment-induced downregulation of MMP-2 and MMP-9 activity and mRNA expression were not linked to phosphorylation of ERK and JNK mainly but only p38. Therefore, it could be suggested that possible inhibition of MMP-2 and MMP-9 by main components of* L. tetragonum* was exerted through TGF-*β* pathways as well as enzymatic inhibition. The LTEs were able to downregulate the MAPK pathway and suggested to possess potential compounds that can inhibit MMP enzymatic activity as well. All data were in accordance with previous findings [13], stating the suggested anti-MMP effects of crude* L. tetragonum* extracts were rooted from the possible bioactive substances from the most active fractions, n-BuOH and 85% aq. MeOH.

**Figure 3:**
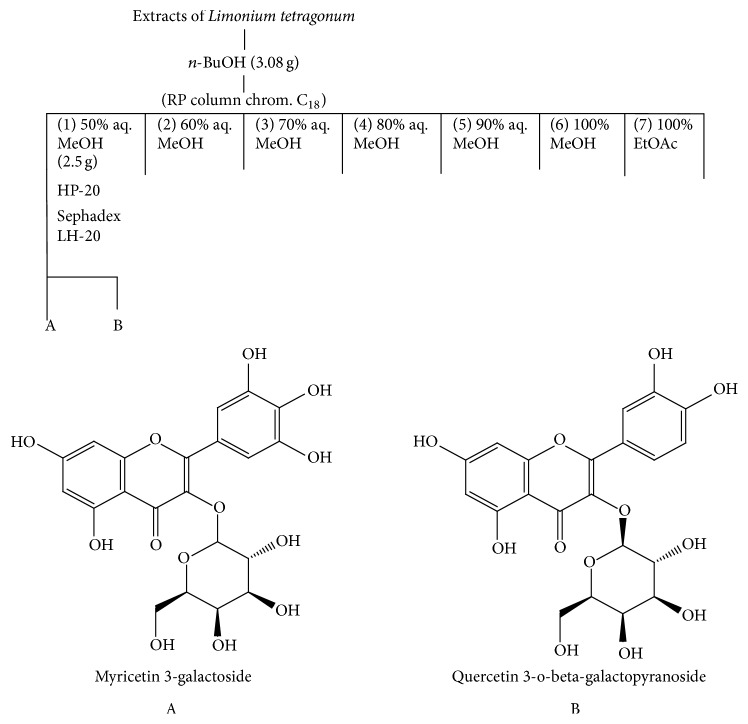
Basic isolation scheme and structural elucidation of two isolated flavonoid glycosides. A: myricetin 3-galactoside; B: quercetin 3-o-beta-galactopyranoside.

Comparing the ability of n-BuOH and 85% aq. MeOH LTEs to inhibit MMP activity and intracellular regulation, two known compounds myricetin 3-galactoside (A) and quercetin 3-o-beta-galactopyranoside (B) were isolated through an activity-guided isolation (Figure 3) as described earlier [14]. These two compounds are known to be derivatives of several antioxidant and anti-MMP compounds which were previously reported [23, 24]. Possible antioxidant properties of these compounds due to their flavonoid backbone were suggested to affect their inhibitory effects on MMPs. Different sizes and chemical structures of the compounds were suggested to be main reason for selective inhibition of MMP activity.

**Figure 4:**
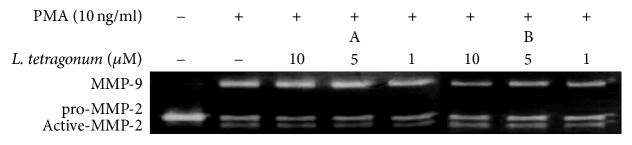
Effects of compounds A and B on enzymatic activity of MMP-2 and MMP-9 tested by gelatin zymography with cell lysates of treated HT1080 fibrosarcoma cells. A: myricetin 3-galactoside; B: quercetin 3-o-beta-galactopyranoside.

**Figure 5:**
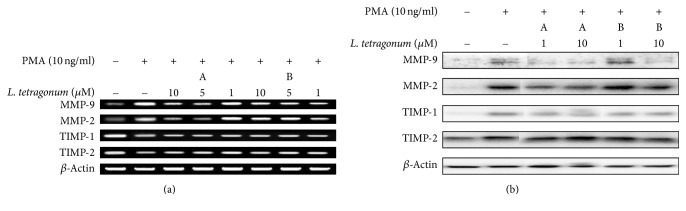
Effects of compounds A and B on mRNA (a) and protein (b) levels of MMP-2 and MMP-9 and TIMP-1 and TIMP-2 observed by RT-PCR and immunoblotting, respectively, in HT1080 human fibrosarcoma cells. *β*-Actin was used as an internal standard. A: myricetin 3-galactoside; B: quercetin 3-o-beta-galactopyranoside.

**Figure 6:**
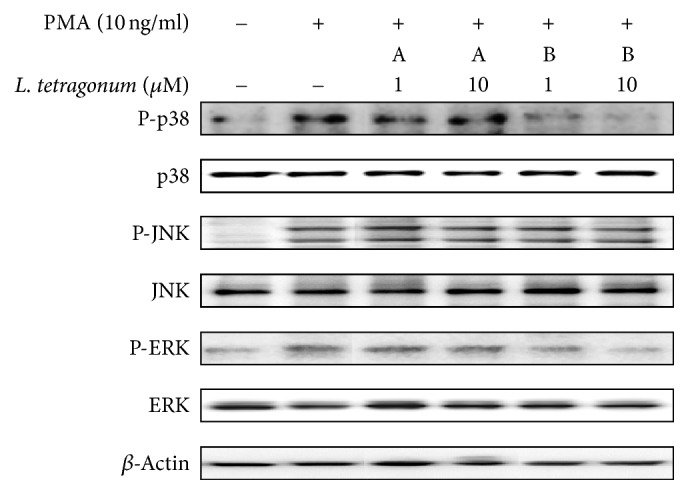
Effect of compounds A and B on phosphorylated (P-) and native protein levels of p38, JNK, and ERK in HT1080 human fibrosarcoma cells. *β*-Actin was used as an internal standard.

In order to verify the compounds as active constituents of the LTEs, their inhibitory effects on MMP activity were tested with gelatin zymography assay (Figure 4). While compound A was able to inhibit MMP-2 activity and fails to affect MMP-9, opposite results were obtained from compound B. Following compound B treatment, MMP-9 activity was lowered up to 63% in a dose-dependent manner while MMP-2 activity was further increased. Further, both compounds were tested for their possible effect on the expression of MMP-2, MMP-9, TIMP-1, and TIMP-2 mRNAs (Figure 5(a)) and proteins (Figure 5(b)). Results confirmed their involvement in regulation of the MMP activity through TIMP-linked pathways. Unlike their effect on enzymatic activity, both compounds were able to lower the PMA-stimulated overexpression of MMP-2 and MMP-9 in a non-dose-dependent manner. Compounds A and B were also able to elevate the PMA-linked downregulation of TIMP-1 and TIMP-2 in terms of mRNA and protein levels. In order to elucidate their action mechanism, compounds were tested for their effect on the levels of MAPK pathway proteins in their preactivation and phosphorylated states. As seen on Figure 6, compound B was able to lower the phosphorylation of ERK and p-38 while compound A showed similar results but not as statistically significant as compound B. Possible intervention of MAPK pathway was hence suggested to be the step in which compound B exerted its anti-MMP activity, confirmed by lowered MMP expression as opposed to slightly affected TIMP levels. Involvement of JNK pathways was suggested to have elevating effects on MMP-2 activity through TIMP-2 regulation. Enhanced MMP-2 activity after compound B treatment can be suggestively linked to its intervention of TIMP-2 and JNK pathways.

## 4. Conclusions

In conclusion,* L. tetragonum* was suggested to contain anti-MMP substances, including but not limited to myricetin 3-galactoside and quercetin 3-o-beta-galactopyranoside that can inhibit MMP activity and suppress MAPK-linked MMP upregulation. Detailed action mechanism of isolated compounds and their efficiency* in vivo* remain unclear. Nonetheless, potential of* L. tetragonum* as a source of anti-MMP compounds for further utilization in nutraceutical and cosmetic fields was verified by current study and paved the way for detailed analysis in order to facilitate the potential of flavonoid glycosides as anti-MMP compounds.

## References

[1] Gialeli C., Theocharis A. D., Karamanos N. K. (2011). Roles of matrix metalloproteinases in cancer progression and their pharmacological targeting. *The FEBS Journal*.

[2] Shuman Moss L. A., Jensen-Taubman S., Stetler-Stevenson W. G. (2012). Matrix metalloproteinases: changing roles in tumor progression and metastasis. *American Society for Investigative Pathology*.

[3] Shay G., Lynch C. C., Fingleton B. (2015). Moving targets: emerging roles for MMPs in cancer progression and metastasis. *Matrix Biology*.

[4] Bourboulia D., Stetler-Stevenson W. G. (2010). Matrix metalloproteinases (MMPs) and tissue inhibitors of metalloproteinases (TIMPs): positive and negative regulators in tumor cell adhesion. *Seminars in Cancer Biology*.

[5] Hsiao W. L. W., Liu L. (2010). The role of traditional Chinese herbal medicines in cancer therapy from TCM theory to mechanistic insights. *Planta Medica*.

[6] Kim S.-K., Thomas N. V. (2010). Metalloproteinase inhibitors: status and scope from marine organisms. *Biochemistry Research International*.

[7] Deryugina E. I., Quigley J. P. (2015). Tumor angiogenesis: MMP-mediated induction of intravasation- and metastasis-sustaining neovasculature. *Matrix Biology*.

[8] Wijesekara I., Kim S.-K., Li Y., Li Y.-X. (2011). Phlorotannins as bioactive agents from brown algae. *Process Biochemistry*.

[9] Liu M., Hansen P. E., Lin X. (2011). Bromophenols in marine algae and their bioactivities. *Marine Drugs*.

[10] Orlikova B., Diederich M. (2012). Power from the garden: plant compounds as inhibitors of the hallmarks of cancer. *Current Medicinal Chemistry*.

[11] Kim N.-H., Sung S. H., Heo J.-D., Jeong E. J. (2015). The extract of Limonium tetragonum protected liver against acute alcohol toxicity by enhancing ethanol metabolism and antioxidant enzyme activities. *Natural Product Sciences*.

[12] Kim J., Kang H., Lee S., Lee J., Park L. (2015). Antioxidant and *α*-glucosidase inhibition activity of seaweed extracts. *Korean Journal of Food Preservation*.

[13] Bae M.-J., Karadeniz F., Lee S.-G., Seo Y., Kong C.-S. (2016). Inhibition of MMP-2 and MMP-9 activities by limonium tetragonum extract. *Preventive Nutrition and Food Science*.

[14] Kwon M. S., Karadeniz F., Kim J.-A., Seo Y., Kong C.-S. (2016). Adipogenesis inhibitory effects of Limonium tetragonum in mouse bone marrow stromal D1 cells. *Food Science and Biotechnology*.

[15] Reuter S., Gupta S. C., Chaturvedi M. M., Aggarwal B. B. (2010). Oxidative stress, inflammation, and cancer: how are they linked?. *Free Radical Biology and Medicine*.

[16] Kuete V., Tankeo S. B., Saeed M. E. M., Wiench B., Tane P., Efferth T. (2014). Cytotoxicity and modes of action of five Cameroonian medicinal plants against multi-factorial drug resistance of tumor cells. *Journal of Ethnopharmacology*.

[17] Khanavi M., Gheidarloo R., Sadati N. (2012). Cytotoxicity of fucosterol containing fraction of marine algae against breast and colon carcinoma cell line. *Pharmacognosy Magazine*.

[18] Wijesekara I., Pangestuti R., Kim S.-K. (2011). Biological activities and potential health benefits of sulfated polysaccharides derived from marine algae. *Carbohydrate Polymers*.

[19] Groblewska M., Mroczko B., Gryko M. (2014). Serum levels and tissue expression of matrix metalloproteinase 2 (MMP-2) and tissue inhibitor of metalloproteinases 2 (TIMP-2) in colorectal cancer patients. *Tumor Biology*.

[20] Reddy K. B., Nabha S. M., Atanaskova N. (2003). Role of MAP kinase in tumor progression and invasion. *Cancer and Metastasis Reviews*.

[21] Davidson B., Givant-Horwitz V., Lazarovici P. (2003). Matrix metalloproteinases (MMP), EMMPRIN (extracellular matrix metalloproteinase inducer) and mitogen-activated protein kinases (MAPK): co-expression in metastatic serous ovarian carcinoma. *Clinical and Experimental Metastasis*.

[22] Kim E.-S., Kim M.-S., Moon A. (2004). TGF-beta-induced upregulation of MMP-2 and MMP-9 depends on p38 MAPK, but not ERK signaling in MCF10A human breast epithelial cells.. *International journal of oncology*.

[23] Vijayababu M. R., Arunkumar A., Kanagaraj P., Venkataraman P., Krishnamoorthy G., Arunakaran J. (2006). Quercetin downregulates matrix metalloproteinases 2 and 9 proteins expression in prostate cancer cells (PC-3). *Molecular and Cellular Biochemistry*.

[24] Lee J. I., Kong C.-S., Eun Jung M., Hong J. W., Lim S. Y., Seo Y. (2011). Antioxidant activity of the halophyte Limonium tetragonum and its major active components. *Biotechnology and Bioprocess Engineering*.

